# Targeting of a polytopic membrane protein to the inner envelope membrane of chloroplasts *in vivo* involves multiple transmembrane segments

**DOI:** 10.1093/jxb/eru290

**Published:** 2014-07-10

**Authors:** Kumiko Okawa, Hitoshi Inoue, Fumi Adachi, Katsuhiro Nakayama, Yasuko Ito-Inaba, Danny J. Schnell, Susumu Uehara, Takehito Inaba

**Affiliations:** ^1^Department of Agricultural and Environmental Sciences, Faculty of Agriculture, University of Miyazaki, Miyazaki 889-2192, Japan; ^2^Department of Biochemistry and Molecular Biology, University of Massachusetts, Amherst, 01003 MA, USA; ^3^Organization for Promotion of Tenure Track, University of Miyazaki, Miyazaki 889-2192, Japan

**Keywords:** *Arabidopsis*, chloroplast, Cor413im1, inner envelope membrane, polytopic proteins, protein targeting.

## Abstract

The inner envelope membrane (IEM) of the chloroplast plays crucial roles in forming an osmotic barrier and controlling metabolite exchange between the organelle and the cytosol. The IEM therefore harbours a number of membrane proteins and requires the import and integration of these nuclear-encoded proteins for its biogenesis. Recent studies have demonstrated that the transmembrane segment of single-spanning IEM proteins plays key roles in determining their IEM localization. However, few studies have focused on the molecular mechanisms by which polytopic membrane proteins are targeted to the IEM. In this study, we investigated the targeting mechanism of polytopic IEM proteins using the protein Cor413im1 as a model substrate. Cor413im1 does not utilize a soluble intermediate for its targeting to the IEM. Furthermore, we show that the putative fifth transmembrane segment of Cor413im1 is necessary for its targeting to the IEM. The C-terminal portion containing this transmembrane segment is also able to deliver Cor413im1 protein to the IEM. However, the fifth transmembrane segment of Cor413im1 itself is insufficient to target a fusion protein to the IEM. These data suggest that the targeting of polytopic membrane proteins to the chloroplast IEM *in vivo* involves multiple transmembrane segments and that chloroplasts have evolved a unique mechanism for the integration of polytopic proteins to the IEM.

## Highlight text

This work demonstrated that the targeting of a polytopic membrane protein to the inner envelope membrane of chloroplasts *in vivo* involves multiple transmembrane segments including the indispensable transmembrane segment.

## Introduction

Plastid biogenesis is dependent on the import of several thousand nuclear-encoded proteins into plastids (Inaba and Schnell, 2008; Jarvis, 2008; Li and Chiu, 2010). The archetypal plastid is the chloroplast found in photosynthetic cells. Chloroplasts consist of six distinct sub-compartments: the outer envelope membrane (OEM), the inner envelope membrane (IEM), stroma, intermembrane space (IMS), thylakoid membrane, and the thylakoid lumen. Each subcompartment plays roles in synthesizing, storing, catabolizing, and transporting metabolites. Hence, the biogenesis and function of each compartment rely on the import and proper suborganelle targeting of nuclear-encoded proteins (Kessler and Schnell, 2009).

Most nuclear-encoded chloroplast proteins are encoded as precursors and harbour an N-terminal transit peptide that is cleaved off after import into the chloroplast (Inaba and Schnell, 2008; Jarvis, 2008; Li and Chiu, 2010). The transit peptide is first recognized by the receptor components of the translocon at the OEM of chloroplasts (TOC) (Andres *et al.*, 2010). Subsequently, the unfolded precursor protein is inserted into the channels of the TOC and the translocon at the IEM (TIC). Translocation of the precursor protein across the TOC-TIC channel is achieved by chaperone activities in the stroma (Flores-Perez and Jarvis, 2013). At the same time, the transit peptide is cleaved by stromal processing peptidase during the translocation. This series of reactions allows precursor proteins to be imported into the chloroplast stroma.

Because the transit peptide only serves as the signal for translocation across envelope membranes, proteins destined to the IEM and thylakoid membranes contain a second, discrete signal that is necessary for their targeting to these locations. Several targeting pathways have been shown to be involved in the targeting of these proteins within the interior of chloroplasts (Celedon and Cline, 2013). Recent studies have demonstrated that IEM proteins utilize two distinct pathways for their targeting to the IEM (Lubeck *et al.*, 1997; Inaba *et al.*, 2005; Li and Schnell, 2006; Tripp *et al.*, 2007; Chiu and Li, 2008; Firlej-Kwoka *et al.*, 2008; Viana *et al.*, 2010; Froehlich and Keegstra, 2011). One is the stop-transfer pathway, and the other is the post-import or conservative pathway. In the stop-transfer pathway, substrate proteins are integrated into the IEM during protein translocation such that they do not utilize soluble intermediates. Substrates of this pathway include ARC6 (Froehlich and Keegstra, 2011), APG1 (Viana *et al.*, 2010), and several other proteins (Chiu and Li, 2008; Firlej-Kwoka *et al.*, 2008). In contrast, substrates of the post-import pathway, such as Tic40, are first targeted to the stroma through the TOC-TIC complex and then re-inserted into the IEM (Li and Schnell, 2006; Tripp *et al.*, 2007). Hence, chloroplasts accumulate a soluble intermediate in the stroma during targeting of the Tic40 protein. The presence of these two pathways has been analyzed in detail using proteins carrying a single transmembrane domain. However, much less is known about the insertion of polytopic IEM proteins. A few studies have suggested that a portion of the polytopic protein may act to provide targeting information to the IEM (Knight and Gray, 1995; Teng *et al.*, 2006; Sattasuk *et al.*, 2011). According to a transient expression assay, the N-terminal half of Tic21 fused to red fluorescent protein (RFP) was delivered to the IEM (Teng *et al.*, 2006). Likewise, the N-terminal portion of a putative amino acid transporter in *Arabidopsis* was sufficient for delivery to the IEM *in vitro* (Sattasuk *et al.*, 2011). Although there is additional evidence that a small portion of polytopic IEM proteins may play a role in their targeting (Knight and Gray, 1995), little is known about the targeting mechanism of polytopic membrane proteins to IEM.

In this work, we investigated the mechanism by which a polytopic membrane protein is targeted to the IEM of chloroplasts. To this end, we chose the Cor413im1 protein as the model substrate. Cor413im1 is a cold-regulated protein in plants (Okawa *et al.*, 2008). It is predicted to have several transmembrane helices and has been shown to localize to the IEM of chloroplasts (Okawa *et al.*, 2008). We show that Cor413im1 does not utilize a soluble intermediate for its targeting to the IEM. Furthermore, we demonstrate that targeting of Cor413im1 to the inner envelope membrane of chloroplasts involves multiple transmembrane segments.

## Materials and methods

### Construction of vectors and *Arabidopsis* transformation

For the construction of the *in vitro* import assay, the *pre-COR413IM1* gene was amplified from *Arabidopsis* cDNA by polymerase chain reaction (PCR) using primers that introduced a 5ʹ NcoI site and a 3ʹ XhoI site. The cDNA was inserted into the NcoI and XhoI sites of pET21d. The pre-APG1 and pre-AtTic40 constructs have been described elsewhere (Li and Schnell, 2006; Viana *et al.*, 2010).

For the construction of TP-pA-Cor413im1 and TP-pA, the transit peptide plus the first five residues of the mature portions of Cor413im1 was amplified by PCR and subcloned into the NcoI and EcoRI sites of pET21d-TEV-pA to generate pET21d-TP-pA. The mature portion of Cor413im1 (amino acids 77–225) was then inserted into the C-terminus of staphylococcal protein A (pA) to create pET-TP-pA-Cor413im1. These plasmids were then digested with NcoI and XbaI and the inserts subcloned into the NcoI and XbaI sites of pCAMBIA3300.

For the construction of a C-terminal deletion series (K30, K50, E73, R95, and K124), a portion of the *pre-COR413IM1* gene was first subcloned into the NcoI and EcoRI sites of pET21d-TEV-pA. The predicted transmembrane helices of Cor413im1 protein and positions of each deletion site are shown in Supplementary Figure S1 (available at *JXB* online). The resulting pET vectors were then digested with NcoI and XbaI and the insert subcloned into the NcoI and XbaI sites of pCAMBIA3300. The pre-Cor413im1-pA construct has been described elsewhere (Okawa *et al.*, 2008).

For the construction of K50-C and R95-C, a portion of the *COR413IM1* gene was subcloned into the C-terminus of pA in pET21d-TP-pA. The resulting pET vectors were then digested with NcoI and XbaI and the insert subcloned into the NcoI and XbaI sites of pCAMBIA3300.

For the construction of TM5-C and K124-C, a portion of the *COR413IM1* gene was subcloned into the C-terminus of pA in pET21d-TP-pA using In-Fusion HD Cloning Kit (Takara). The resulting insert, TP-pA-TM5 and TP-pA-K124, was further amplified by PCR, and the amplified fragment was subcloned into the NcoI and XbaI sites of pCAMBIA3300 using In-Fusion HD Cloning Kit.

All pCAMBIA constructs were introduced into *Arabidopsis thaliana* (accession Columbia) via *Agrobacterium tumefaciens*-mediated transformation by the floral dip method (Clough and Bent, 1998).

### *Arabidopsis* chloroplast isolation and membrane fractionation

For chloroplast isolation, *Arabidopsis* plants were grown on 0.5x MS plates supplemented with 1% sucrose at 23°C for 2 weeks. Chloroplasts were isolated from 14- to 18-day-old transgenic plants expressing each chimeric protein as described previously (Smith *et al.*, 2002).

For the preparation of total membrane and soluble proteins, isolated chloroplasts were diluted in either 0.2M Na_2_CO_3_ (pH 12.0), or 1% Triton X-100, and incubated for 10min on ice. The samples were then separated into soluble and membrane fractions by ultracentrifugation at 100 000 *g* for 15min.

### Analysis of the localization of truncated proteins within chloroplasts

To determine the localization of truncated proteins within chloroplasts, isolated chloroplasts were fractionated into stroma, envelope, and thylakoid membranes as described previously (Smith *et al.*, 2002). After the quantification of proteins in each fraction, total chloroplast (3 μg), stroma (3 μg), envelope (1 μg), and thylakoid (1.5 μg) fractions were analyzed by sodium dodecyl sulfate polyacrylamide gel electrophoresis (SDS-PAGE) and immunoblotting with the antisera indicated in the figures. Although we sometimes loaded different amount of proteins for the analysis, the protein ratio of total chloroplast:stroma:envelope:thylakoid was always 3:3:1:1.5. The trypsin sensitivity of Cor413im1-pA was examined using intact chloroplasts, as described previously (Jackson *et al.*, 1998; Li and Schnell, 2006).

Antibodies against Tic110 (Inaba *et al.*, 2005) and ACCase (Kozaki *et al.*, 2000) have already been described. Rabbit polyclonal antibody against Toc75 was produced using Toc75_(141–397)_-His as the antigen based on previous literature (Hiltbrunner *et al.*, 2001). The anti-protein A IgG was purchased from Sigma-Aldrich.

The fold enrichment of each truncated protein in the envelope fraction was estimated as follows. The signal intensity of each chimeric protein band in total chloroplast and envelope fractions was first measured by densitometric software (CS analyzer, ATTO) and the signal intensity per microgram protein was calculated based on the amount of protein loaded. Then, the value for the envelope fraction was divided by the value for the total chloroplast fraction to estimate the fold enrichment in the envelope fraction compared to the total chloroplast. As the positive control, we also estimated fold enrichment of Tic110 protein in the envelope fraction.

### *In vitro* translation and protein import assay

[^35^S]Met-labelled wild-type and mutant preCor413im1 proteins were generated in a coupled transcription-translation system containing reticulocyte lysate according to the manufacturer’s instructions (Promega, Madison, WI, USA). Time-course import assays were performed using intact pea chloroplasts as described previously (Viana *et al.*, 2010). The quantitative analysis of radiolabeled samples was performed with a Typhoon FLA7000 PhosphorImager and ImageQuant TL software (GE Healthcare Life Sciences).

## Results

### Cor413im1 does not utilize a soluble intermediate for its integration into the chloroplast inner envelope

**Fig. 1.**
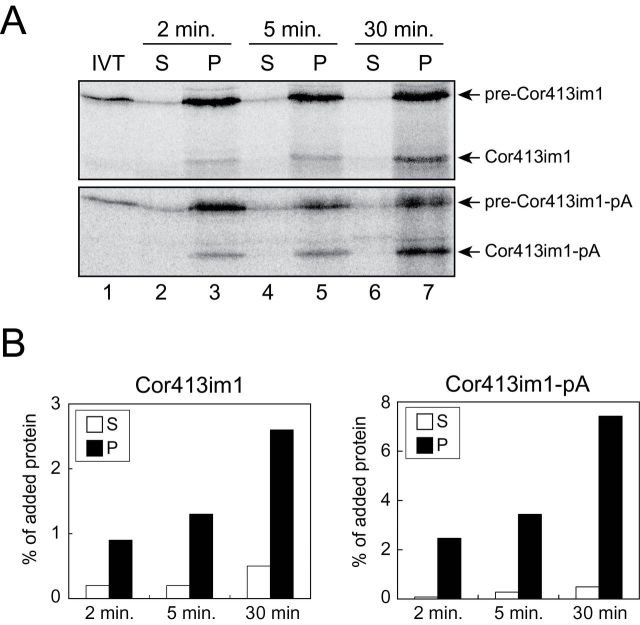
Cor413im1 does not utilize a soluble intermediate during targeting to the IEM. (A) [^35^S]pre-Cor413im1 (upper panel) or [^35^S]pre-Cor413im1-pA (lower panel) was imported into chloroplasts for the times indicated in the presence of 5mM ATP. The reactions were stopped by the addition of an excess of HEPES-sorbitol buffer. The chloroplasts were then lysed and separated into soluble (S) and membrane (P) fractions. IVT, *in vitro* translation product. (B) The graphs represent the quantification of lanes 2–7 for mature Cor413im1 or mature Cor413im1-pA.

We first examined the pathway Cor413im utilizes for its integration into the chloroplast inner envelope. To this end, we conducted a time-course import experiment using *in vitro*-translated ^35^S-labelled pre-Cor413im1. According to previous studies, substrates for the stop-transfer pathway do not utilize soluble intermediates (Chiu and Li, 2008; Firlej-Kwoka *et al.*, 2008; Viana *et al.*, 2010; Froehlich and Keegstra, 2011). In contrast, precursors that use the post-import pathway, such as Tic40, have been shown to accumulate soluble intermediates during the early time points in the import assay (int-atTic40 in Supplementary Figure S2, available at *JXB* online). We took this approach to discriminate between these two pathways. Aliquots were taken from the import reaction mixture at each time point and added into excess ice-cold HEPES-sorbitol buffer to stop the import reaction. The samples were then fractionated into the membrane versus soluble fractions. As shown in Fig. 1A, B, mature Cor413im1 accumulated in the membrane fraction in a time-dependent manner. In contrast, we did not observe a detectable amount of soluble Cor413im1 (Fig. 1A, B). This observation was further confirmed by the *in vitro* import assay using protein A-tagged pre-Cor413im1 (pre-Cor413im1-pA in Fig. 1). Both Cor413im1 and Cor413im1-pA proteins were also resistant to thermolysin treatment in intact chloroplasts (Supplementary Figure S2, available at *JXB* online), indicating that they were imported into the chloroplast interior. The distribution of Cor413im1 and Cor413im1-pA is quite similar to that of APG1, a known substrate of the stop-transfer pathway (Supplementary Figure S2, available at *JXB* online). These results indicate that Cor413im1 does not utilize a soluble intermediate for its integration into the chloroplast inner envelope.

### The mature portion of Cor413im1 contains information necessary for its targeting to the IEM

**Fig. 2.**
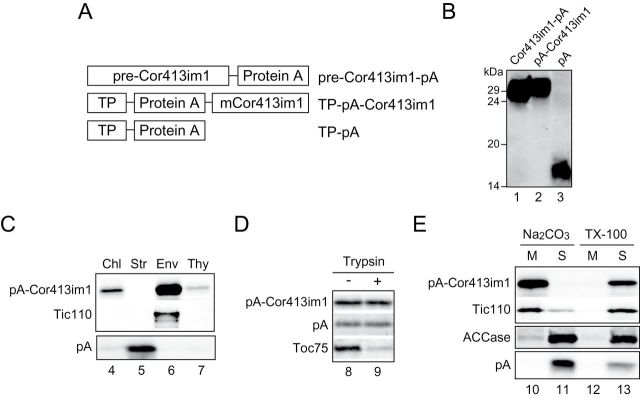
The mature portion of Cor413im1 contains the information necessary for targeting to the IEM. (A) Schematic diagram of chimeric Cor413im1 constructs used in these studies. The pre-Cor413im1-pA was previously shown to correctly target to the chloroplast IEM. (B) Expression analysis of chimeric proteins in transgenic *Arabidopsis*. Total protein extracts from true leaves were resolved by SDS-PAGE and immunoblotted using an antibody to protein A. (C) Localization of pA-Cor413im1 and pA in chloroplasts. Isolated chloroplasts (Chl) were fractionated into stroma (Str), envelope (Env), and thylakoid (Thy) fractions. The protein ratio of Chl:Str:Env:Thy used in the analysis was 3:3:1:1.5. Each fraction was resolved by SDS-PAGE and immunoblotted with antibodies against protein A (pA-Cor413im1 and pA) or Tic110. (D) Trypsin sensitivity of chimeric Cor413im1 proteins in intact chloroplasts. Chloroplasts equivalent to 25 µg chlorophyll were treated with trypsin on ice for 30min. The trypsin was inactivated and intact chloroplasts were re-isolated, resolved by SDS-PAGE, and immunoblotted with antibody against protein A. The protease sensitivity of the OEM protein, Toc75, was included as a control for trypsin activity. (E) Localization of pA-Cor413im1 and pA in the soluble and membrane fractions of chloroplasts. Chloroplasts were lysed in the buffer containing 0.2M Na_2_CO_3_, pH 12 (Na_2_CO_3_) or 1% Triton X-100 (TX-100) and separated into membrane (M) and soluble (S) fractions. The extracts were resolved by SDS-PAGE and immunoblotted with antibodies against protein A (pA-Cor413im1 and pA), ACCase CTα subunit, or Tic110.

It has been shown that the mature portion plays a key role in the integration of IEM proteins into the membrane, regardless of the pathways they use. This proposal is based on the analysis of IEM proteins that have either one or two transmembrane domains (Li and Schnell, 2006; Tripp *et al.*, 2007; Viana *et al.*, 2010; Froehlich and Keegstra, 2011). However, much less is known about polytopic transmembrane proteins. Therefore, we next investigated the role of the transit peptide and the mature portions in the integration of Cor413im1 into the IEM. We generated constructs in which the transit peptide of Cor413im1 was fused to the amino terminus of protein A (Fig. 2A, TP-pA). In one construct, we also fused the mature portion of Cor413im1 to the C-terminus of TP-pA (Fig. 2A, TP-pA-Cor413im1). We previously demonstrated that the pre-Cor413im1-pA (Fig. 2A) was properly localized to the IEM (Okawa *et al.*, 2008). These three constructs were transformed into *Arabidopsis* and the expressed proteins were detected by immunoblotting (Fig. 2B). When chloroplasts isolated from *Arabidopsis* expressing TP-pA-Cor413im1 were further fractionated into stroma, envelope, and thylakoid fractions, pA-Cor413im1 signals were enriched in the envelope fraction (Fig. 2C). The pA-Cor413im1 was resistant to trypsin treatment of intact chloroplasts (Fig. 2D), indicating that the expressed proteins were targeted to the interior of the IEM. It was also shown that pA-Cor413im1 was resistant to Na_2_CO_3_ extraction but can be solubilized by Triton X-100 (Fig. 2E, lanes 10–13). This localization was similar to Cor413im1-pA (Okawa *et al.*, 2008) as well as the inner envelope protein Tic110 (Fig. 2C, 2E). We also confirmed that the peripherally associated IEM protein, acetyl CoA carboxylase (ACCase) (Sasaki *et al.*, 1993; Shorrosh *et al.*, 1996; Nakayama *et al.*, 2007), could be extracted by Na_2_CO_3_ treatment (Fig. 2E), indicating that pA-Cor413im1 is an integral membrane protein of the IEM. In contrast, signals from TP-pA plants were observed in the stroma before (Fig. 2C, pA) and after (Fig. 2E, pA) Na_2_CO_3_ extraction. These results indicate that the transit peptide portion of Cor413im1 acts as the targeting signal into the stroma. Furthermore, the mature portion of Cor413im1 contains the information necessary for its targeting to the IEM.

### Identification of a specific transmembrane segment necessary for the efficient integration of Cor413im1 into the IEM

**Fig. 3.**
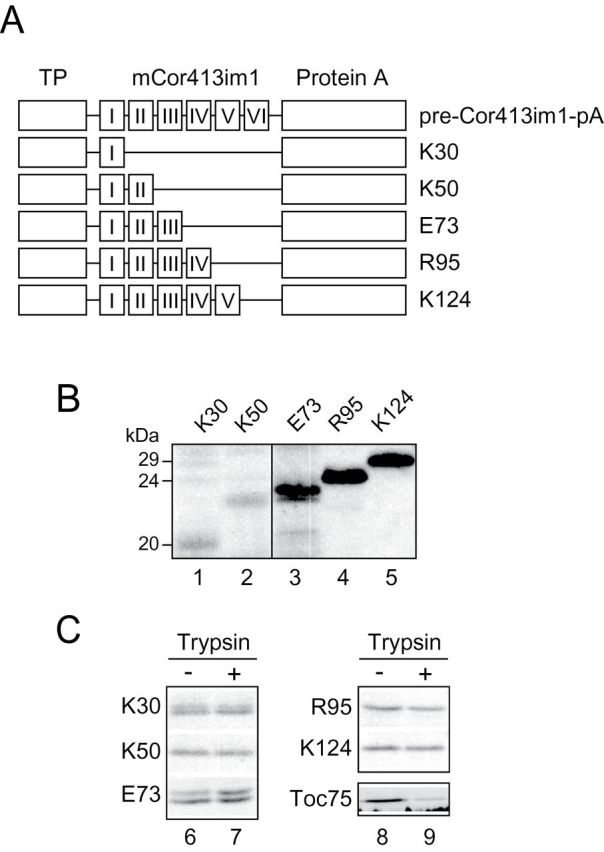
Expression of C-terminal truncated Cor413im1 proteins in transgenic *Arabidopsis*. (A) Schematic diagram of Cor413im1 C-terminal deletion constructs. Roman numerals indicate the predicted positions of each transmembrane segment. (B) Expression analysis of C-terminal truncated Cor413im1 proteins in transgenic *Arabidopsis*. Total proteins were extracted from true leaves, resolved by SDS-PAGE and immunoblotted with antibodies against protein A to detect chimeric proteins. (C) Trypsin sensitivity of C-terminal truncated Cor413im1 proteins in intact chloroplasts. Chloroplasts equivalent to 25 µg chlorophyll were treated with trypsin on ice for 30min. The trypsin was inactivated and intact chloroplasts were re-isolated, resolved by SDS-PAGE, and immunoblotted with antibody against protein A. The protease sensitivity of the OEM protein, Toc75, was included as a control for trypsin activity.

Cor413im1 is predicted to contain six potential transmembrane segments (Breton *et al.*, 2003; Okawa *et al.*, 2008). Therefore, we generated a series of deletion constructs carrying one to five transmembrane segments fused to protein A and investigated their localization within the chloroplast (Fig. 3A and Supplementary Figure S1, available at *JXB* online). These constructs were transformed into *Arabidopsis* and the protein expression was confirmed by immunoblotting (Fig. 3B). When intact chloroplasts isolated from these plants were treated with trypsin, all of the expressed proteins were resistant to trypsin digestion (Fig. 3C), indicating that the expressed proteins were targeted to the interior of the IEM. The control outer envelope protein, Toc75, was digested by trypsin, indicating that the protease was active (Fig. 3C).

**Fig. 4.**
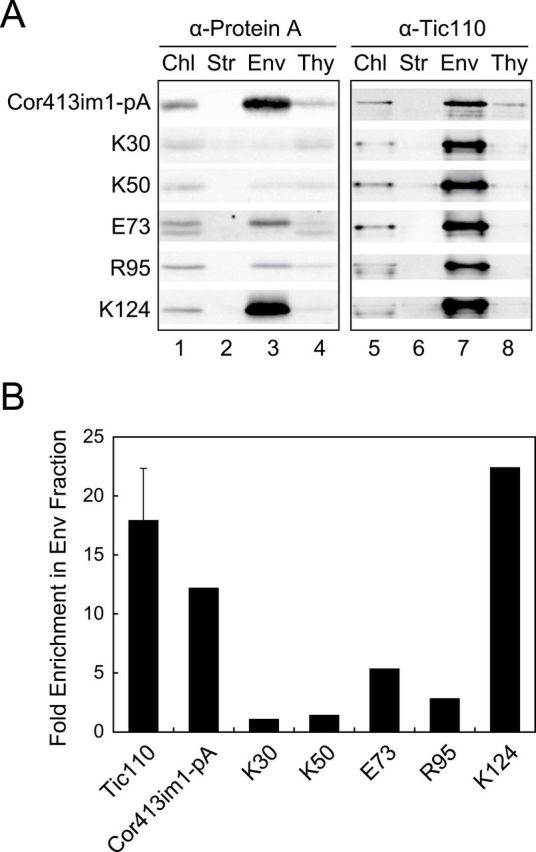
Identification of the transmembrane segment necessary for the targeting of Cor413im1 to the IEM. (A) Localization of C-terminal truncated Cor413im1 chimeric proteins within chloroplasts. Isolated chloroplasts (Chl) were fractionated into stroma (Str), envelope (Env), and thylakoid (Thy) fractions. Each fraction was resolved by SDS-PAGE, and immunoblotted with antibodies against protein A or Tic110. The protein ratio of Chl:Str:Env:Thy used in these analyses was always 3:3:1:1.5. (B) Fold enrichment of each truncated protein in the envelope fraction. The signal intensity of each band (lanes 1 and 3) was first measured by densitometry and the signal intensity per microgram protein was calculated based on the amount of protein loaded. Then, the value for the envelope fraction was divided by the value for the total chloroplast fraction to estimate the fold enrichment in the envelope fraction compared to the total chloroplast. As a control, average fold enrichment of Tic110 protein in the envelope fraction was also calculated. For Tic110, lanes 5 and 7 were used to measure signal intensity. Because we used 12.5% resolving gel for SDS-PAGE in Fig. 4, the fold enrichment of Tic110 and other envelope-localized proteins is higher than those found in Fig. 7.

We further fractionated chloroplasts into stroma, envelope, and thylakoid fractions and investigated the localization of the truncated proteins (Fig. 4A). To assess the IEM localization of each chimeric protein, we quantified the signal intensity per microgram of protein of each band in the total chloroplast and envelope fractions and estimated the fold enrichment of each protein in the envelope fraction. When we used the 12.5% acrylamide resolving gel for this analysis, the average fold enrichment of Tic110, a marker protein of the IEM, was ~15 (Fig. 4B). Consistent with the previous observation, the full-length chimeric protein, Cor413im1-pA, was also enriched more than 10 times (Fig. 4B). When the fold enrichment of each expressed protein was estimated, the enrichment of K124 was as good as those of Cor413im1-pA and Tic110 (Fig. 4B). In contrast, other chimeric proteins, K30, K50, E73, and R95, were not enriched in the envelope fraction efficiently (Fig. 4B).

**Fig. 5.**
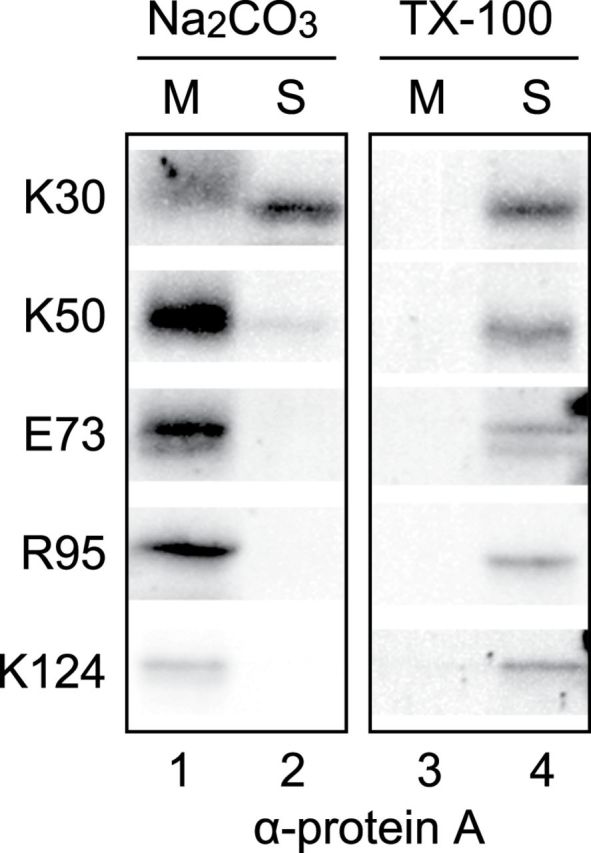
Membrane association of C-terminal truncated Cor413im1 proteins. Localization of C-terminal truncated Cor413im1 proteins in soluble and membrane fractions of chloroplasts. Chloroplasts were lysed in buffer containing 0.2M Na_2_CO_3_, pH 12 (Na_2_CO_3_), or 1% Triton X-100 (TX-100) and separated into membrane (M) and soluble (S) fractions. The extracts were resolved by SDS-PAGE and immunoblotted with antibodies against protein A.

To investigate whether the chimeric proteins were integrated into the membrane or associated with the membrane peripherally, chloroplasts were lysed with Na_2_CO_3_ and fractionated into the membrane and soluble fractions. As shown in Fig. 5, the K30 protein was partitioned into both the soluble and membrane fractions. However, most of the other truncated proteins were exclusively localized in the membrane fraction and could be solubilized by Triton X-100 (Fig. 5).

Collectively, these data suggest that the fifth transmembrane segment play crucial roles in the efficient targeting of Cor413im1 to the IEM. Furthermore, it seems that a portion of K30, K50, E73 and R95 was mistargeted and integrated at the thylakoid membranes instead of the IEM.

### Multiple transmembrane segments containing the fifth transmembrane segment are indispensable for the targeting of Cor413im1 to the IEM

**Fig. 6.**
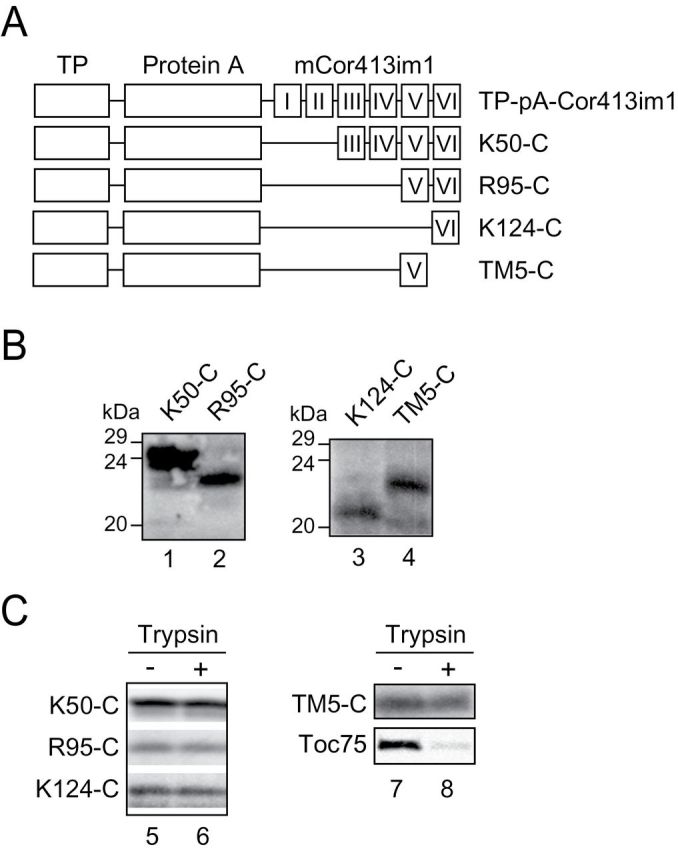
Expression of N-terminal truncated Cor413im1 proteins in transgenic *Arabidopsis*. (A) Schematic diagram of N-terminal truncated Cor413im1 constructs. Roman numerals indicate the predicted positions of each transmembrane segment. (B) Accumulation of N-terminal truncated Cor413im1 protein in *Arabidopsis*. Total proteins were extracted from true leaves, resolved by SDS-PAGE, and immunoblotted using antibodies against protein A to detect chimeric proteins. (C) Trypsin sensitivity of the N-terminal truncated Cor413im1 proteins in intact chloroplasts. Chloroplasts equivalent to 25 µg of chlorophyll were treated with trypsin on ice for 30min. Intact chloroplasts were re-isolated, resolved by SDS-PAGE, and immunoblotted with antibody against protein A. The protease sensitivity of the OEM protein, Toc75, was included as a control for trypsin activity.

Given our results indicating that the deletion of the C-terminus resulted in the inefficient targeting of Cor413im1 proteins to the envelope membrane, we wished to demonstrate whether the C-terminal portion is sufficient to deliver chimeric proteins to the IEM. To this end, we generated amino-terminal deletion constructs of Cor413im1 and fused them to the TP-pA construct (Fig. 6A). These chimeric constructs contain the transit peptide and the C-terminal portion of mature Cor413im1, but lack the N-terminal portion (Fig. 6A).

**Fig. 7.**
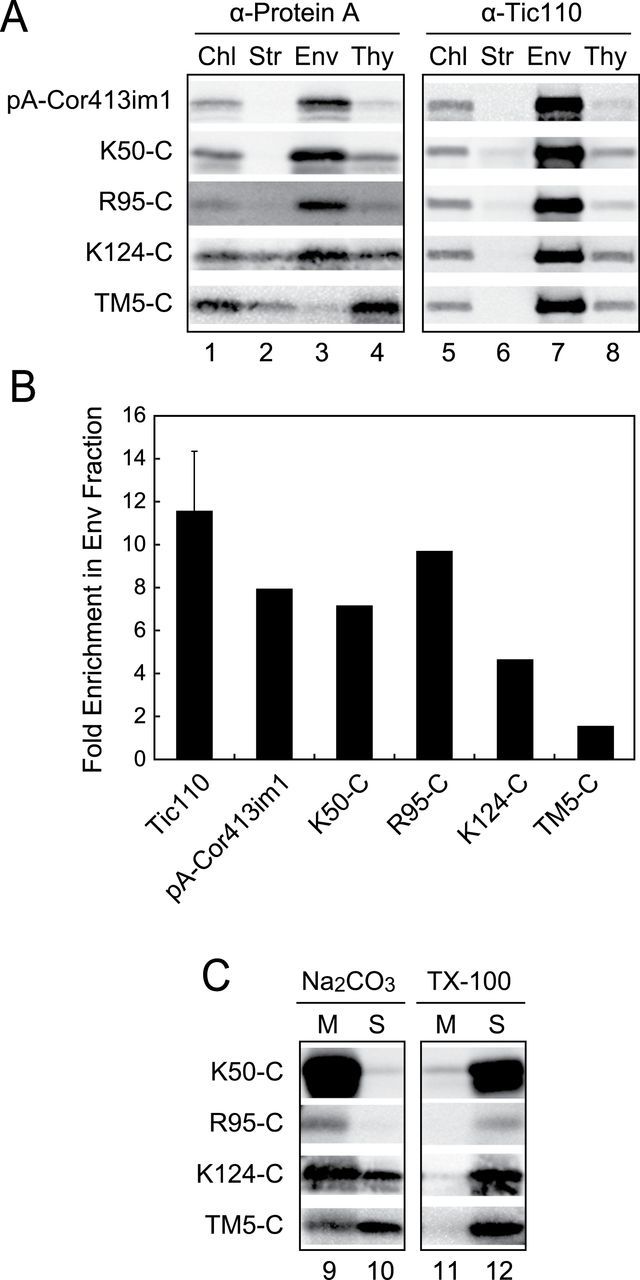
Targeting of Cor413im1 to the chloroplast IEM involves multiple transmembrane segments containing the fifth transmembrane segment. (A) Localization of the N-terminal truncated Cor413im1 proteins within chloroplasts. Isolated chloroplasts (Chl) were fractionated into stroma (Str), envelope (Env), and thylakoid (Thy) fractions. Each fraction was resolved by SDS-PAGE, and immunoblotted with the antibodies against protein A or Tic110. The protein ratio of Chl:Str:Env:Thy used in these analyses was always 3:3:1:1.5. (B) Fold enrichment of each truncated protein in the envelope fraction. The signal intensity of each band [lanes 1 and 3 in panel (A)] was first measured by densitometry and the signal intensity per microgram protein was calculated based on the amount of protein loaded. Then, the value for the envelope fraction was divided by the value for the total chloroplast fraction to estimate the fold enrichment in the envelope fraction. As a control, average fold enrichment of Tic110 protein in the envelope fraction was also calculated. For Tic110, lanes 5 and 7 in panel (A) were used to measure signal intensity. Because we used 5–20% acrylamide gradient gel for SDS-PAGE in Fig. 7, the fold enrichment of Tic110 and other envelope-localized proteins is lower than those found in Fig. 4. (C) Localization of N-terminal truncated Cor413im1 proteins in the soluble and membrane fractions of chloroplasts. Chloroplasts were lysed in the buffer containing 0.2M Na_2_CO_3_, pH 12 (Na_2_CO_3_), or 1% Triton X-100 (TX-100) and separated into membrane (M) and soluble (S) fractions. The extracts were resolved by SDS-PAGE and immunoblotted with antibodies against protein A.

The three chimeric proteins, K50-C, R95-C and K124-C, were stably expressed in transgenic *Arabidopsis* (Fig. 6B). When chloroplasts isolated from transgenic plants were treated with trypsin, all the proteins examined were resistant to trypsin treatment (Fig. 6C, left panel), indicating that they were imported into the interior of the IEM. We further fractionated chloroplasts into the stroma, envelope, and thylakoid fractions. When each fraction was resolved by 5–20% acrylamide gradient gel in SDS-PAGE and immunoblotting, K50-C and R95-C proteins were enriched in the envelope fraction and their distribution was similar to those of Tic110 and pA-Cor413im1 (Fig. 7A, B). These proteins were also resistant to Na_2_CO_3_ extraction (Fig. 7C). In contrast, K124-C protein failed to be targeted to the envelope membrane efficiently (Fig. 7A, B). Furthermore, K124-C was extractable from the membrane with Na_2_CO_3_, indicating that the protein was not properly integrated into the membrane. Again, these data indicate that the fifth transmembrane segment is necessary for the targeting of Cor413im1 to the IEM *in vivo*.

We next investigated if the fifth transmembrane segment is sufficient for the targeting to the IEM. To this end, we constructed a chimeric gene consisting of the fifth transmembrane segment and TP-pA (Fig 6A, TM5-C). The TM5-C protein was expressed in transgenic *Arabidopsis* (Fig 6B, lane 4). Furthermore, TM5-C was imported into the interior of the IEM *in vivo* (Fig. 6C, right panel) as it was resistant to trypsin treatment of intact chloroplasts. We next fractionated chloroplasts into the stroma, envelope, and thylakoid fractions. As shown in Fig. 7A, B, TM5-C was no longer enriched in the envelope fraction. Instead, we observed strong signal in the thylakoid fraction. The protein was extracted by Na_2_CO_3_ (Fig. 7C), indicating that TM5-C was not properly integrated into the chloroplast membrane.

Taken together, we conclude that the fifth transmembrane segment is necessary for the targeting of Cor413im1 to the IEM. However, the fifth transmembrane segment itself is insufficient to target fusion proteins to the IEM. Instead, multiple transmembrane segments containing the fifth transmembrane segment are indispensable for the efficient targeting of Cor413im1 to the IEM.

## Discussion

The plastid IEM plays key roles in synthesizing, catabolizing, and transporting metabolites. However, the biogenesis of IEM proteins, especially polytopic IEM proteins, remains to be characterized in detail. In this study, we aimed to investigate how polytopic proteins are targeted to the IEM. We chose Cor413im1 as the model substrate and showed that Cor413im1 does not utilize a soluble intermediate for its targeting to the IEM. This suggests that Cor413im1 most likely utilizes the stop-transfer pathway for its integration to the IEM. Furthermore, we also provide evidence that the small portion of Cor413im1 plays crucial roles in determining IEM localization *in vivo*.

We observed that C-terminal truncated Cor413im1 proteins that lack the fifth transmembrane segment were not efficiently enriched into the IEM (Fig. 4A, B), but were resistant to Na_2_CO_3_ extraction (Fig. 5). This suggests that a portion of truncated Cor413im1 was mislocalized to the thylakoid membrane. Indeed, TM5-C protein carrying insufficient targeting information to the IEM was also mislocalized to the thylakoid membrane (Fig. 7A). This implies that hydrophobic proteins lacking the IEM targeting signal are targeted to thylakoid membranes once they are imported into chloroplasts. We speculate that the thylakoid membrane is the default destination for hydrophobic membrane proteins within chloroplasts if they do not have the IEM insertion signal. Another possible explanation is that the truncated forms of Cor413im1 lacking the IEM targeting signal cannot discriminate the IEM from the thylakoid membrane. The thylakoid membrane is far more abundant than the IEM within the chloroplast. Hence, unless membrane proteins have an additional signal to direct them to the IEM, they predominantly localize to the thylakoid membrane. The second hypothesis is consistent with the observation that some truncated proteins lacking the IEM targeting signal were also found in the envelope fraction (e.g. E73 and R95 in Fig. 4; and K124-C in Fig.7). Our results reemphasize that the IEM targeting signal is indispensable for the biogenesis of polytopic IEM proteins.

Both C-terminal (K124 in Fig. 4) and N-terminal (K50-C and R95-C in Fig. 7) truncated Cor413im1 containing the fifth transmembrane segment were integrated into the IEM. However, the fifth transmembrane segment itself is insufficient for efficient targeting to the IEM (TM5-C in Fig. 7). This result implies that the presence of multiple transmembrane segments is indispensable for the integration of polytopic membrane proteins to the IEM. A similar observation was made for targeting of the polytopic phosphate translocator to the chloroplast IEM (Knight and Gray, 1995). When the putative first transmembrane segment of the phosphate translocator (Willey *et al.*, 1991) was fused to the small subunit of ribulose bisphosphate carboxylase/oxygenase, the majority of chimeric proteins were detected in the soluble fraction (Knight and Gray, 1995). Our results also raise the possibility that the targeting pathway of polytopic IEM proteins is distinct from that of monotopic IEM proteins. This hypothesis is supported by the fact that the transmembrane segment derived from monotopic protein, such as APG1 and ARC6, is sufficient to integrate chimeric proteins to IEM in *in vitro* import assays (Viana *et al.*, 2010; Froehlich and Keegstra, 2011).

In mitochondria, the targeting mechanism of polytopic inner membrane proteins is distinct from that of presequence-carrying precursors. The TOM complex harbours distinct receptors for each class of proteins (Becker *et al.*, 2012). Furthermore, they also have the specialized TIM complex, the TIM22 complex, for the integration of polytopic proteins from the intermembrane space to the inner membrane (Jensen and Dunn, 2002). In chloroplasts, it appears that discrimination between abundant photosynthetic proteins and less-abundant non-photosynthetic proteins are mediated by multiple TOC receptors (Inaba and Schnell, 2008; Jarvis, 2008; Kessler and Schnell, 2009; Li and Chiu, 2010). Although it remains unclear whether the specialized pathway for polytopic membrane proteins exists, the hypothesis that chloroplasts contain specialized translocation machinery for polytopic proteins is consistent with our observations.

Froehlich and Keegstra suggested that the transmembrane segments of monotopic IEM proteins are rich in aromatic amino acids and lack proline residues relative to thylakoid proteins (Froehlich and Keegstra, 2011). However, the requirement of multiple transmembrane segments for IEM targeting of polytopic membrane proteins (Figs 4 and 7) will make definition of the physical characteristics of any IEM targeting signal difficult to define. In mitochondria, polytopic proteins are inserted into the inner membrane from intermembrane space by the TIM22 complex (Jensen and Dunn, 2002). During their targeting, a module consisting of two helices connected by a matrix-facing loop serves as a signal for integration into inner membrane (Davis *et al.*, 1998). Therefore, requirement of multiple transmembrane segments is reminiscent of polytopic protein targeting in mitochondrial inner membrane. However, this mechanism also requires the membrane potential across inner mitochondrial membrane (Ferramosca and Zara, 2013). Unlike inner mitochondrial membrane, the chloroplast IEM lacks a significant membrane potential. Furthermore, polytopic IEM proteins also possess a cleavable transit peptide that is not usually found in mitochondrial carrier proteins (Inaba and Schnell, 2008; Ferramosca and Zara, 2013). Hence, polytopic chloroplast IEM proteins are unlikely to utilize the same mechanism as mitochondrial inner membrane proteins. Rather, we hypothesize that additional IEM components participate in the integration of polytopic IEM proteins. A recent study identified a second type of Sec machinery, cpSec2, which is predominantly localized in the IEM (Skalitzky *et al.*, 2011). The level of cpSec2 is co-regulated with the levels of Tic40 and Tic110 (Celedon and Cline, 2013). Hence, it is intriguing to hypothesize that cpSec2 and TIC machinery cooperate to act as a membrane integrase for polytopic IEM proteins that is targeted by the stop-transfer mechanism (Celedon and Cline, 2013). If this is the case, it is possible that the separate transmembrane segments are distinctively recognized by Tic and Sec machineries such that multiple transmembrane segments are required for efficient targeting of polytopic proteins to the IEM. The cpSec might also help the flanking transmembrane segments at the stromal side (e.g. TM1-TM4 of translocating Cor413im1) avoid aggregation during translocation through the TIC channel.

According to our analysis, the protein A portion of K124 is resistant to trypsin treatment (Fig. 3C). Because K124 lacks the sixth transmembrane segment, the topology of K124 seems to be reverted compared to Cor413im1-pA (Okawa *et al.*, 2008). These data suggest that the IEM-targeting signal of polytopic proteins plays key roles in precise targeting to the IEM but does not determine the transmembrane topology. A previous study suggested that IEP37 is flipped at the IEM (Viana *et al.*, 2010), suggesting the presence of a topology-flipping mechanism at the IEM. Therefore, it is plausible to speculate that some of the truncated proteins, such as K124, are subjected to this regulation. Furthermore, in contrast to the transgenic study (Fig. 7C), we found that *in vitro* imported K124-C and TM5-C proteins were fractionated into the membrane fraction after Na_2_CO_3_ extraction (Supplementary Figure S3, available at *JXB* online). This suggests that the *in vitro* import assay using isolated chloroplasts could not completely reconstitute the targeting of truncated Cor413im1 proteins observed *in vivo*. Chloroplasts appear to regulate the biogenesis of polytopic IEM proteins at multiple levels.

It appears that chloroplasts have evolved stop-transfer and post-import/conservative IEM protein targeting systems that are distinct from those in mitochondria. In mitochondria, Oxa1p, a homologue of bacterial YidC in mitochondria, participates in the insertion of membrane proteins from mitochondrial matrix via a conservative pathway (Wang and Dalbey, 2011). However, in chloroplasts, the YidC homologue, Alb3, is relocated to the thylakoid membrane (Moore *et al.*, 2000), and there is no evidence that YidC/Oxa1/Alb3 homologues exist on the IEM. Identification of the components mediating the chloroplast IEM-targeting pathways will shed additional light on the unique mechanisms of inner membrane biogenesis in this organelle.

## Supplementary material

Supplementary data can be found at *JXB* online.

Supplementary Figure S1. A putative secondary structural model of *Arabidopsis* Cor413im1 protein.

Supplementary Figure S2. Distribution of Cor413im1, Cor413im1-pA, APG1, int-atTic40, and atTic40 after thermolysin treatment of intact chloroplasts.

Supplementary Figure S3. *In vitro* protein import assay of truncated Cor413im1 proteins.

## Funding

This work was supported by the Programme to Disseminate Tenure Tracking System from the Japanese Ministry of Education, Culture, Sports, Science, and Technology, a grant for Scientific Research on Priority Areas from the University of Miyazaki (to TI and YII), Naito Foundation, Strategic Young Researcher Overseas Visits Programme for Accelerating Brain Circulation, Grant-in-Aid for Young Scientists (B, no. 25850073) to TI, and a National Institutes of Health Grant 2RO1-GM061893 (to DJS). HI was a recipient of a Japan Society for the Promotion of Science postdoctoral fellowship for research abroad.

## Supplementary Material

Supplementary Data

## Abbreviations:

IMS: intermembrane space of the chloroplast envelope
TOC: translocon at the outer membrane of chloroplasts
TIC: translocon at the inner membrane of chloroplasts
IEM: inner envelope membrane
OEM: outer envelope membrane
RFP: red fluorescent protein
PCR: polymerase chain reaction
SDS-PAGE: sodium dodecyl sulfate polyacrylamide gel electrophoresis.
