# Supplementary material for: Targeting of a polytopic membrane protein to the inner envelope membrane of chloroplasts in vivo involves multiple transmembrane segments
Source: J Exp Bot. 2014 Jul 10;65(18):5257–65. doi: 10.1093/jxb/eru290 (PMC4157711; doi:10.1093/jxb/eru290)
Supplement: Supplementary Data [file supp_65_18_5257__index.html]

Targeting of a polytopic membrane protein to the inner envelope membrane of chloroplasts in vivo involves multiple transmembrane segments — Targeting of a polytopic membrane protein to the inner envelope membrane of chloroplasts in vivo involves multiple transmembrane segments — Supplementary Data 

# Targeting of a polytopic membrane protein to the inner envelope membrane of chloroplasts *in vivo* involves multiple transmembrane segments

## Supplementary Data

Data files

**Files in this Data Supplement:**

- Supplementary Data - Supplementary Data
