# Supplementary material for: Targeting of a polytopic membrane protein to the inner envelope membrane of chloroplasts in vivo involves multiple transmembrane segments
Source: J Exp Bot. 2014 Jul 10;65(18):5257–65. doi: 10.1093/jxb/eru290 (PMC4157711; doi:10.1093/jxb/eru290)
Supplement: Supplementary Data [file supp_eru290_jexbot123984_file001.pdf]

Targeting of a polytopic membrane protein to the inner envelope membrane of chloroplasts *in vivo* involves multiple transmembrane segments

Kumiko Okawa, Hitoshi Inoue, Fumi Adachi, Katsuhiro Nakayama, Yasuko Ito-Inaba,

Danny J. Schnell, Susumu Uehara and Takehito Inaba

Supplementary materials

Figs S1-S3

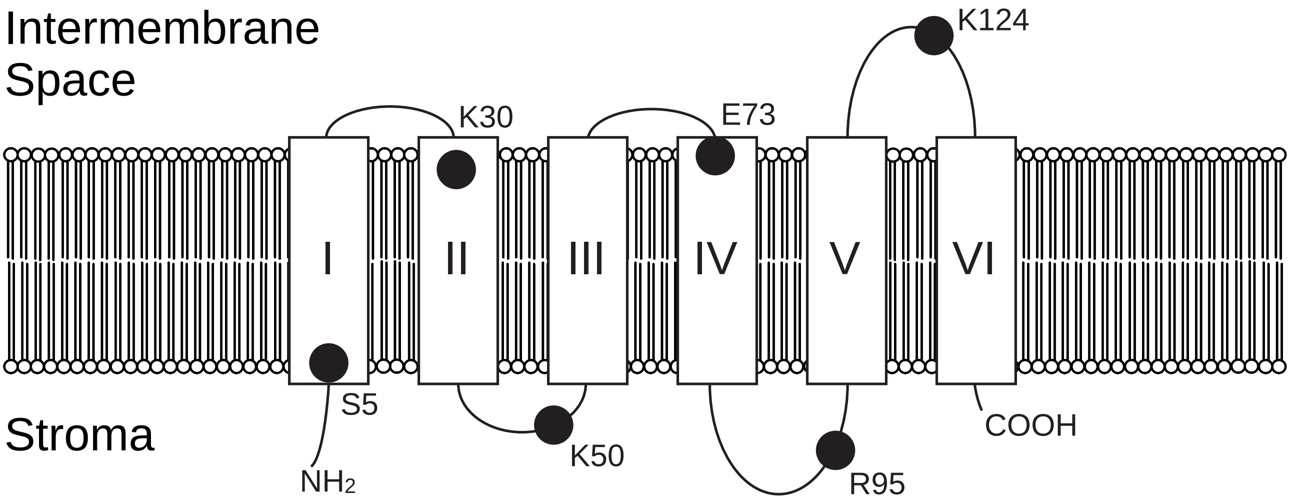

Figure S1. A putative secondary structural model of Arabidopsis Cor413im1 protein.
The putative transmembrane segments of mature Cor413im1 were predicted by ConPredII (*Nucleic Acids Res.*, 32: W390-3). The amino terminus of mature Cor413im1 was experimentally determined in the previous study (*Plant Cell Environ.*, 31, 1470-83). Boxes numbered with Roman numerals represent the putative 6 transmembrane helices. Position of Cor413im1 truncation sites in this study is indicated by filled circles with the single letter amino acid code followed by the residue number.

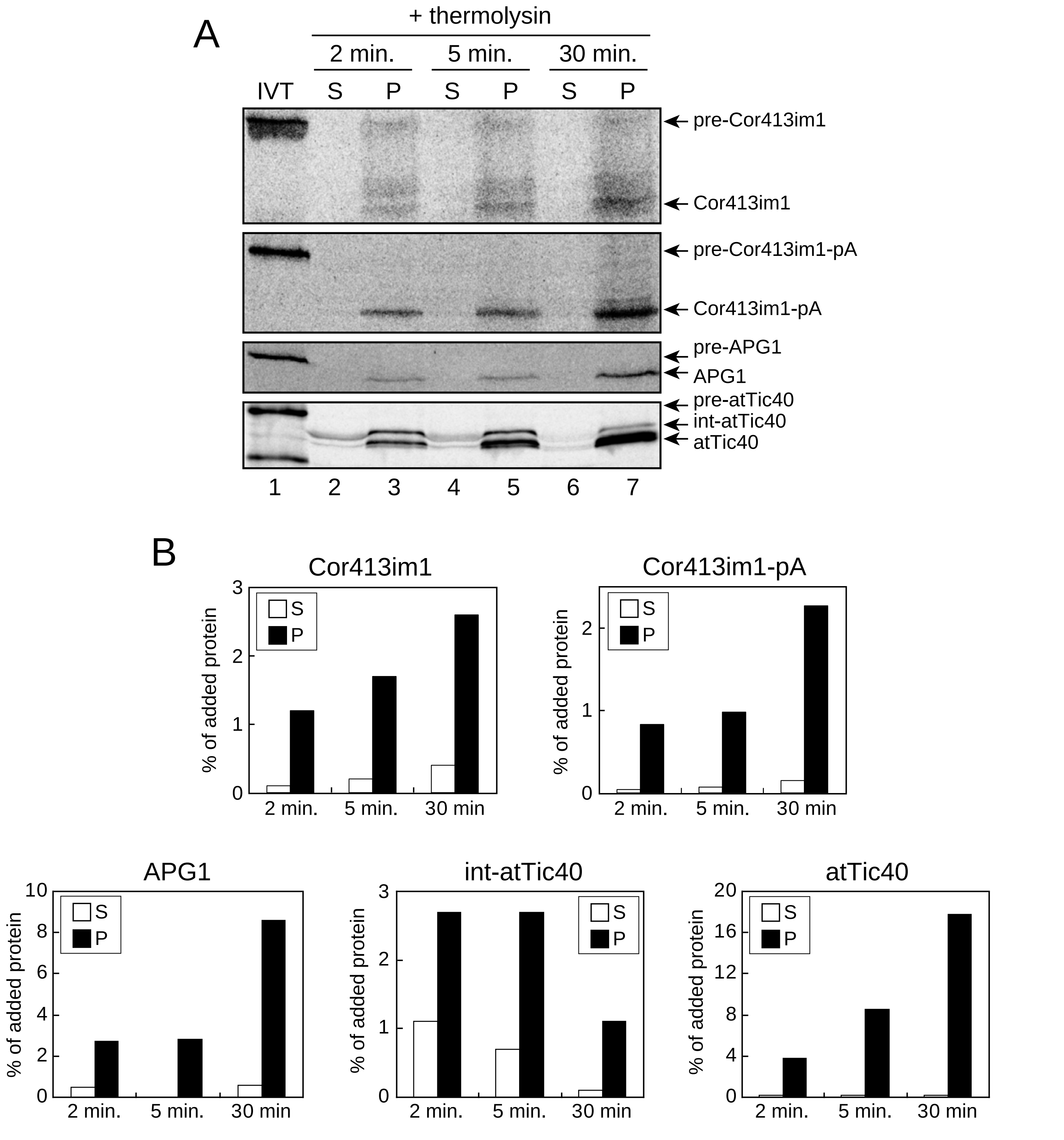

Figure S2. Distribution of Cor413im1, Cor413im1-pA, APG1, int-atTic40 and atTic40 after thermolysin treatment of intact chloroplasts.

A, [^{35}S]pre-Cor413im1 (first panel), [^{35}S]pre-Cor413im1-pA (second panel), [^{35}S]pre-APG1 (third panel), or [^{35}S]pre-atTic40 (fourth panel) was imported into chloroplasts for the times indicated in the presence of 5 mM ATP. The reactions were stopped by the addition of an excess of HEPES-sorbitol buffer, and the chloroplasts were incubated in the presence of thermolysin (200 μg/ml) on ice for 30 min. After the proteolysis was stopped, the chloroplasts were lysed and separated into soluble (S) and membrane (P) fractions. IVT, *in vitro* translation product.

B, The graphs represent the quantification of lanes 2–7 for mature Cor413im1, mature Cor413im1-pA, mature APG1, intermediate atTic40 (int-Tic40), or mature atTic40.

Okawa et al.
Figure S3

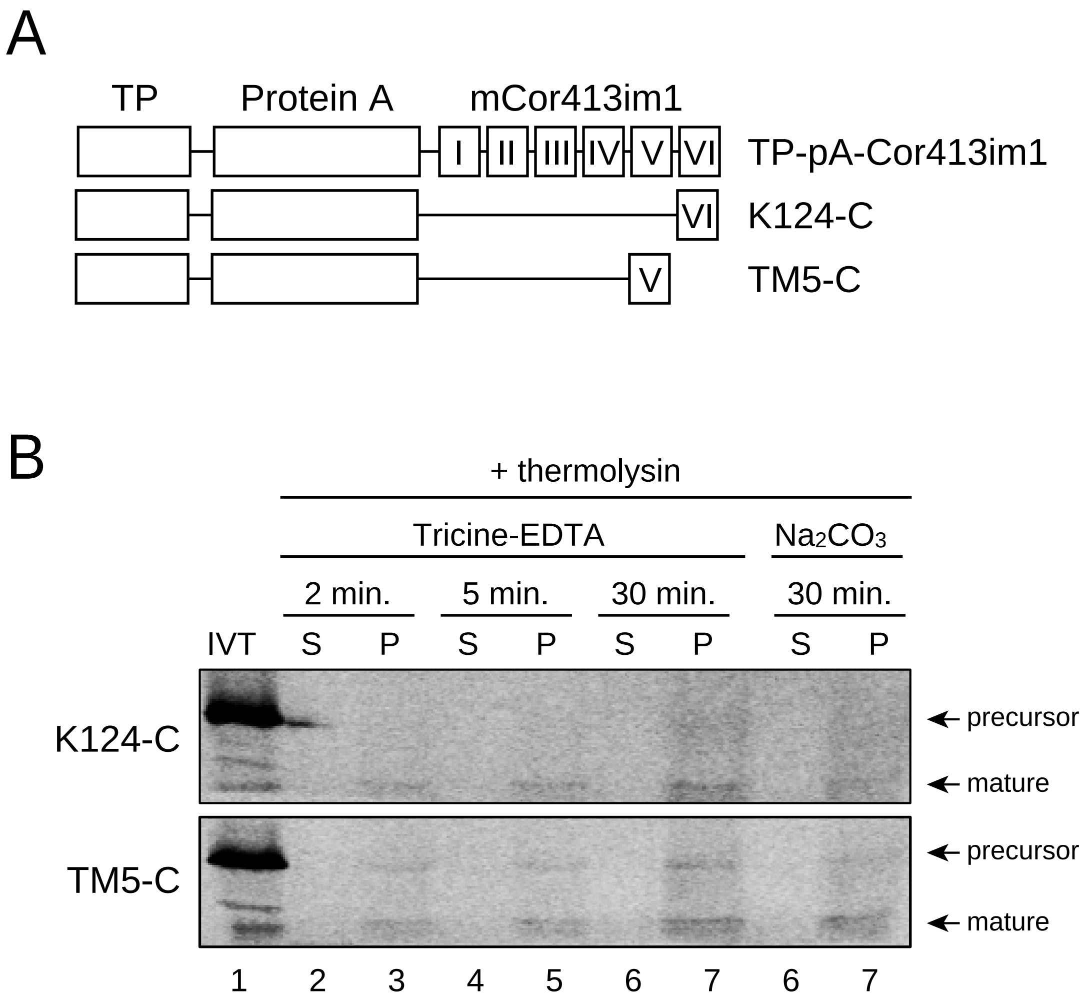

Figure S3. ***In vitro*** **protein import assay of truncated Cor413im1 proteins.**
A, Schematic diagram of truncated Cor413im1 constructs used in this assay. Roman numerals indicate the predicted positions of each transmembrane segment.
B, [^{35}S]K124-C (upper panel) or [^{35}S]TM5-C (lower panel) was imported into chloroplasts for the times indicated in the presence of 5 mM ATP. The reactions were stopped by the addition of an excess of HEPES-sorbitol buffer, and the chloroplasts were incubated in the presence of thermolysin (200 μg/ml) on ice for 30 min. After the proteolysis was stopped, the chloroplasts were lysed and separated into soluble (S) and membrane (P) fractions. IVT, *in vitro* translation product.
